# PSO-FeatureFusion: a general framework for fusing heterogeneous features via particle swarm optimization

**DOI:** 10.1093/bioadv/vbaf263

**Published:** 2025-10-29

**Authors:** Raziyeh Masumshah, Changiz Eslahchi

**Affiliations:** School of Biological Sciences, Institute for Research in Fundamental Sciences (IPM), Tehran 193955746, Iran; School of Biological Sciences, Institute for Research in Fundamental Sciences (IPM), Tehran 193955746, Iran

## Abstract

**Motivation:**

Integrating heterogeneous biological data is a central challenge in bioinformatics, especially when modeling complex relationships among entities such as drugs, diseases, and molecular features. Existing methods often rely on static or separate feature extraction processes, which may fail to capture interactions across diverse feature types and reduce predictive accuracy.

**Results:**

To address these limitations, we propose PSO-FeatureFusion, a unified framework that combines particle swarm optimization with neural networks to jointly integrate and optimize features from multiple biological entities. By modeling pairwise feature interactions and learning their optimal contributions, the framework captures individual feature signals and their interdependencies in a task-agnostic and modular manner. We applied PSO-FeatureFusion to two bioinformatics tasks—drug-drug interaction and drug-disease association prediction—using multiple benchmark datasets. Across both tasks, the framework achieved strong performance across evaluation metrics, often outperforming or matching state-of-the-art baselines, including deep learning and graph-based models. The method also demonstrated robustness with limited hyperparameter tuning and flexibility across datasets with varying feature structures. PSO-FeatureFusion provides a scalable and practical solution for researchers working with high-dimensional biological data. Its adaptability and interpretability make it well-suited for applications in drug discovery, disease prediction, and other bioinformatics domains.

**Availability and implementation:**

The source code and datasets are available at https://github.com/raziyehmasumshah/PSO-FeatureFusion.

## 1 Introduction

Bioinformatics integrates biology, computer science, mathematics, and statistics to develop computational tools for analyzing complex biological datasets such as genomic, proteomic, drug, disease, and interaction data (Searls 2000, Pevsner 2015, Ritchie *et al.* 2015, Xia 2017, Bhat *et al.* 2022, Singh and Singh 2024). For example, genomic data may include gene expression patterns or genetic variants, while proteomic data focus on protein sequences and structures. Drug-related data typically describe molecular characteristics or side effects, while disease-related data consist of biomarkers or clinical observations. Interaction data capture relationships between biological entities, such as protein-protein or drug-disease interactions. These heterogeneous datasets offer unique perspectives on biological systems but also pose significant challenges in terms of feature extraction and integration, particularly due to issues such as dimensional variability, semantic heterogeneity, and data sparsity (Masumshah *et al.* 2021, Jia *et al.* 2023, Masumshah and Eslahchi 2023, Hassanali Aragh *et al.* 2024, He *et al.* 2024, Lenhof *et al.* 2024, Meng *et al.* 2024, Shi *et al.* 2024, Svedberg *et al.* 2024, Taylor *et al.*. 2024, Wang *et al.* 2024, Wu *et al.* 2024, Zhang *et al.* 2024, Zhu *et al.* 2024). Through feature extraction and integration, researchers can simplify complex biological data, improve interpretability, and build robust models for tasks in genomics, proteomics, drug discovery, and disease diagnosis (Al-Rabeah and Lakizadeh 2022, Hassanali Aragh *et al.* 2024, Kobraei *et al.* 2024). Feature extraction involves identifying the most informative characteristics of biological entities, while feature integration combines them to support deeper understanding and predictive modeling of the underlying biological mechanisms. Feature extraction methods generate entity-specific representations tailored to each biological domain, but to fully understand biological systems, these features must be integrated. Feature integration enables a more comprehensive view of the relationships among entities and is essential for developing accurate predictive models. To provide a clearer view of the state-of-the-art, we organize the existing bioinformatics methods for feature extraction into six distinct categories: (i) similarity-based methods, (ii) network-based methods, (iii) matrix factorization-based methods, (iv) machine learning-based methods, (v) ensemble learning-based methods, and (vi) deep learning-based methods. In the following, we review representative methods from each category, highlighting their core mechanisms and limitations, and laying the foundation for positioning our proposed approach within the broader context of bioinformatics integration strategies.

- Similarity-based methods: These methods compute similarity scores between entities (e.g. drugs, diseases, genes) based on predefined features and use these scores for downstream predictions. For example, Hassanali Aragh *et al.* (2024) calculated drug and disease pair similarities to enhance drug repositioning efforts. Similarly, the DPSP method combined drug-related features such as side effects, target proteins, and biochemical pathways to improve drug-drug interaction prediction (Masumshah and Eslahchi 2023). Peng *et al.* (2023) introduced MHCLMDA, which uses similarity-based hypergraphs and autoencoders to integrate miRNA and disease features for miRNA-disease association prediction.
- Network-based methods: These approaches model biological entities and interactions as graphs and use graph-based algorithms to discover hidden relationships. Zitnik *et al.* (2018) developed Decagon, a multimodal graph convolutional model for predicting polypharmacy side effects. Zeng *et al.*. (2024) proposed GNNGL-PPI, which integrates global and local subgraph features for protein-protein interaction prediction.
- Matrix factorization-based methods: Matrix factorization techniques decompose biological interaction matrices (e.g. drug-disease or drug-target) into latent factors, enabling prediction of missing links. Gao *et al.* (2023) proposed MDA-AENMF, which uses autoencoders with non-negative matrix factorization for metabolite–disease association prediction. Ha (2022) introduced MDMF, a method that incorporates disease similarity constraints into matrix factorization to improve miRNA-disease association prediction. However, these methods often assume linear relationships and may not capture complex interaction patterns in biological systems.
- Machine learning-based methods: Machine learning methods further extend predictive capabilities by employing supervised, unsupervised, or reinforcement learning algorithms to analyze complex, heterogeneous datasets. Kim *et al.* (2019) predicted drug-disease associations using multiple similarity features and classification models such as logistic regression and random forests. Mei and Zhang (2021) used L2-regularized logistic regression for drug interaction prediction based on drug target profiles. Ding *et al.* (2020) introduced the RWLR model, which uses a random walk with restart on circRNA-circRNA similarity networks to infer circRNA-disease associations.
- Ensemble learning-based methods: Ensemble learning methods improve prediction robustness by combining multiple machine learning models, often yielding better generalization than individual models. Cheng and Zhao (2014) integrated naive Bayes, decision trees, k-nearest neighbors, logistic regression, and support vector machines to enhance drug-drug interaction prediction. Kastrin *et al.* (2018) approached the same problem as a link prediction task, leveraging k-nearest neighbors to infer potential drug interactions within networks. Although these methods are efficient and scalable, they often treat feature integration as a separate, static preprocessing step and may not learn complex feature dependencies.
- Deep learning-based methods: Deep learning models offer powerful representation learning capabilities for biological data. Wei *et al.* (2024) employed a self-supervised 2D convolutional neural network to recover missing gene expression data. Gao *et al.* (2024) introduced AutoDDI, an automated graph neural network (GNN) design for predicting drug-drug interactions. Yuan *et al.* (2022) used graph convolutional networks to predict protein–protein interaction sites. Elbasani *et al.* (2021) developed a compound-protein interaction model combining GNNs and RNNs. Jiang *et al.* (2022) demonstrated the utility of transformers in DeepTTA, which predicts anti-cancer drug responses using transcriptomic data. Although effective, these models usually rely on concatenation-based feature fusion and may not dynamically learn optimal interactions between different types of features.

Despite these advancements, a key limitation shared across most existing methods is the lack of dynamic modeling for feature interdependencies. Feature integration is typically handled through static techniques such as concatenation, summation, or inner product, which may overlook intricate, task-specific interactions between features of different biological entities (Feng *et al.* 2020). Furthermore, many methods require manual feature engineering or are sensitive to data sparsity and dimensional mismatches. To address these limitations, we propose PSO-FeatureFusion, a novel framework that utilizes particle swarm optimization (PSO) to dynamically discover optimal combinations of feature representations. This method models complex interfeature relationships between biological entities while preserving their individual characteristics. It is flexible, generalizable across tasks, and demonstrates strong performance on multiple bioinformatics challenges, including polypharmacy side effect prediction and drug-disease association discovery. Specifically, PSO-FeatureFusion addresses key challenges in biological data modeling as follows:

- Data sparsity: Data sparsity is mitigated by transforming raw features into similarity matrices, generating denser and more informative representations. The integration of heterogeneous biological features further helps compensate for missing or incomplete information.
- Feature dimensional mismatch: Feature dimensional mismatch is handled by applying dimensionality reduction techniques such as PCA or autoencoders, ensuring standardized and compatible feature representations across entities.
- Computational inefficiency: Computational inefficiency is addressed through a modular and parallelizable design. Each feature pair is modeled using a lightweight neural network, and PSO optimizes their contributions without requiring heavy end-to-end training.

Through this design, PSO-FeatureFusion offers a scalable and effective solution for modeling complex biological systems by dynamically capturing inter-feature dependencies. In the following sections, we describe the architecture and optimization procedure in detail, and demonstrate the framework’s effectiveness across two key prediction tasks: polypharmacy side effect prediction and drug–disease association discovery.

## 2 Methods

**Figure 1.**
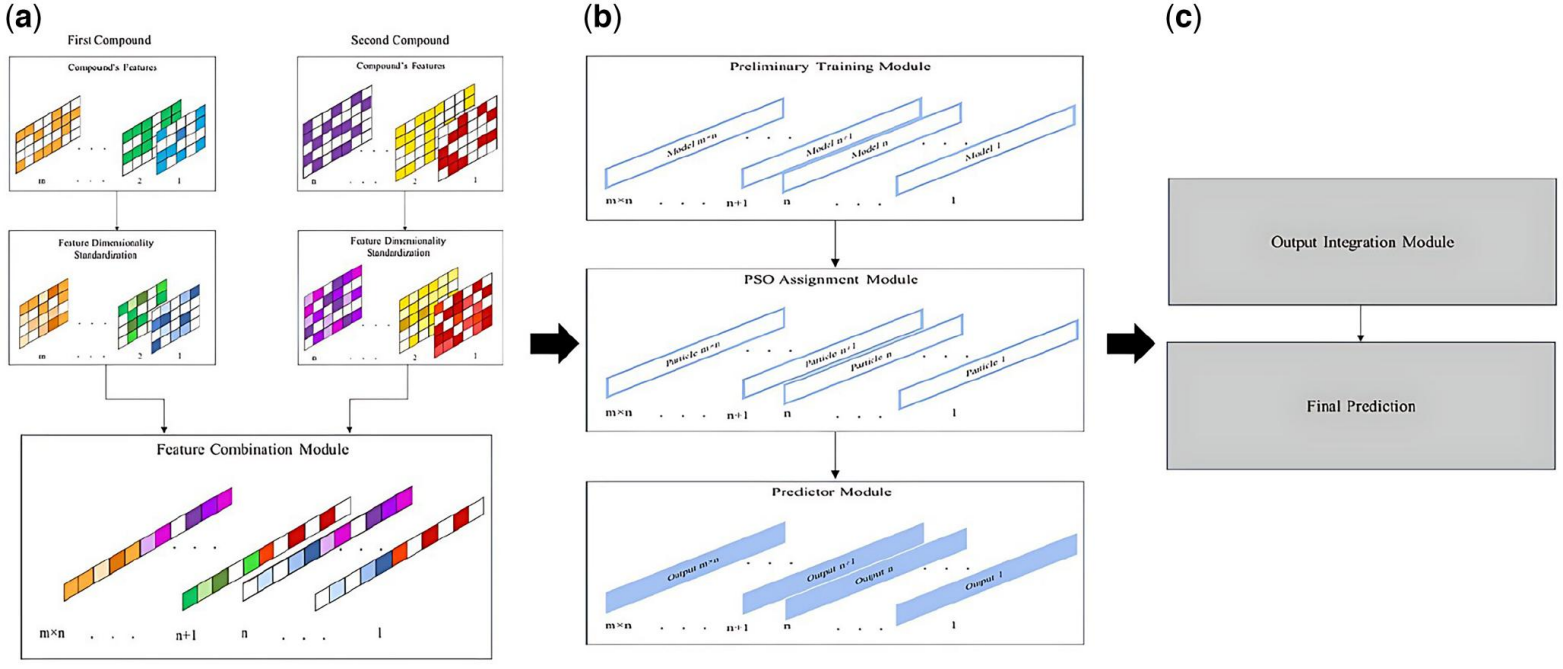
(a) Construction and combination of input features to create meaningful representations. (b) Training individual models, optimizing assignments using the PSO algorithm, and generating predictions via the preliminary training, PSO-based assignment, and prediction modules. (c) Integration of outputs from individual models using the output integration module, followed by the final prediction module to produce the ultimate predictions.

In this section, we introduce PSO-FeatureFusion, a predictive framework designed to model complex relationships between two biological entities, A and B, in a variety of tasks such as link prediction, classification, and regression. The framework aims to overcome limitations associated with handling heterogeneous feature sets and modeling interactions between entities. To achieve this, PSO-FeatureFusion follows a three-step pipeline: (i) feature preparation and combination, in which features from both entities are standardized and integrated; (ii) model training and optimization, where particle swarm optimization (PSO) is used to fine-tune predictive models; and (iii) output integration and final prediction, where results from multiple models are aggregated into a robust final output. The framework is specifically designed to address challenges like feature dimensional mismatches, sparse data, and complex entity interactions, thereby enhancing predictive accuracy and generalizability. An overview of the architecture is illustrated in Fig. 1.

### 2.1 Feature preparation and combination

This phase ensures that the features of entities A and B are standardized and systematically combined to capture their interactions. It includes two key steps: feature dimensionality standardization and feature combination.

#### 2.1.1 Feature dimensionality standardization

The dataset consists of two entities:

- A set A of size *k*, denoted as $\left\{a_{1},a_{2},\ldots ,a_{k}\right\}$, with *n* features $\left\{f_{1},f_{2},\ldots ,f_{n}\right\}$ per element.
- A set B of size *l*, denoted as $\left\{b_{1},b_{2},\ldots ,b_{l}\right\}$, with *m* features $\left\{F_{1},F_{2},\ldots ,F_{m}\right\}$ per element.

To address the variability in feature dimensions (*n* and *m*), we apply the following standardization procedure:

1. Similarity matrix construction:
  - For each feature $f_{k}$ where k=1,…,n in A, a similarity matrix of size k×k is computed, quantifying pairwise relationships between elements in A. This results in n similarity matrices for A.
  - Similarly, *m* similarity matrices are generated for entity B, each of size l×l.
  - Each row of a similarity matrix is treated as a feature vector, thereby representing entity elements in a consistent form.
2. Dimensionality reduction:
  - To unify these vectors into a lower-dimensional representation while preserving important patterns, dimensionality reduction techniques such as principal component analysis (PCA) or autoencoders are applied.
  - The resulting standardized features are used in subsequent modeling steps (Fig. 1a).

While various dimensionality reduction techniques, such as principal component analysis (PCA) and autoencoder-based methods, can be used to map heterogeneous feature sets into a unified lower-dimensional space, in this study, we primarily adopted a similarity-based dimensionality standardization approach. Specifically, for each feature set, we computed pairwise similarity matrices using Jaccard similarity to convert sparse and high-dimensional feature vectors into dense, uniformly structured representations. Based on preliminary experiments, this method consistently outperformed PCA and autoencoder-based techniques in terms of predictive performance and computational efficiency. Consequently, it was selected as the default standardization method in our framework.

#### 2.1.2 Feature combination

Once standardized, features from entities A and B are systematically paired to model interactions:

- For every pair $\left(a_{i},b_{j}\right)$, their corresponding feature vectors are concatenated to form a combined feature vector.
- This process generates m×n unique feature combinations, each representing a specific interaction between one feature from A and one from B.
- These combined feature vectors serve as the input to individual predictive models in the training and optimization phase (Fig. 1a).

### 2.2 Model training and optimization

This phase focuses on training individual models for each feature pair and optimizing their contributions using the particle swarm optimization (PSO) algorithm. It includes three key stages: preliminary training, PSO assignment and optimization, and output integration and final prediction.

#### 2.2.1 Preliminary training

Each combined feature pair generated in the previous step is assigned to a dedicated predictive model aimed at learning the interaction between corresponding features from entities A and B. Given m×n feature pairs, the framework creates m×n predictive models. To ensure fairness and isolate the effect of feature combinations, all models share the same neural network architecture and hyperparameters (Fig. 1b).

- The models are trained independently using a fixed number of epochs. Training proceeds sequentially across models—each one is trained before moving to the next.
- This pre-training phase establishes a consistent baseline performance across models, preparing them for PSO-based optimization.
- The loss function used is task-dependent:
- For regression tasks: mean squared error (MSE).
- For classification tasks: binary cross-entropy, F-measure, or other problem-specific criteria.

#### 2.2.2 PSO assignment and optimization

In this stage, we use the particle swarm optimization (PSO) algorithm to directly optimize the internal parameters (weights and biases) of individual neural networks, following an iterative training-refinement strategy. Each particle in the swarm corresponds to a neural network model associated with a specific feature pair. Initially, all networks are independently trained using standard backpropagation. PSO is then applied iteratively to refine the networks by updating their internal parameters based on both personal best and global best positions within the swarm.

Particle representation: Each particle represents a neural network, and its position encodes the complete set of learnable parameters (i.e. weights and biases).

Number of particles: The swarm consists of m×n particles, corresponding to all combinations of feature pairs (drug–drug for DDI, drug–disease for DDA).

Velocity and position updates: The standard PSO update rules are applied to evolve the internal parameters of each network:


(1) $$v_{i}(t+1)=\omega v_{i}(t)+c_{1}r_{1}(p_{i}(t)-x_{i}(t))+c_{2}r_{2}(p_{g}(t)-x_{i}(t))$$

(2) $$x_{i}(t+1)=x_{i}(t)+v_{i}(t+1)$$

Where:

-
$x_{i}(t)$ : Parameters (weights and biases) of the $i^{th}$ network at iteration *t*
-
$v_{i}(t)$ : Velocity of the parameter update
-
$p_{i}$ : Personal best parameter set of the $i^{th}$ particle
-
$p_{g}$ : Global best parameter set found by the swarm
-
ω : Inertia weight
-
$c_{1},c_{2}$ : Cognitive and social learning coefficients
-
$r_{1},r_{2}$ : Random scalars in [0, 1]

Training after PSO update: After each PSO position update, the corresponding neural network is re-trained using its new parameters as initialization. This re-training phase allows the network to adapt to the new parameter configuration and better fit its assigned feature pair, thereby improving fitness evaluation.

Fitness function: Following re-training, each network is evaluated on a validation set using a task-specific loss function. The resulting loss is used to update the personal and global bests of the PSO process.

#### 2.2.3 PSO dynamics

1. Initialization:
  - All networks are first independently trained and evaluated.
  - Their parameters are used as the initial positions in the swarm.
2. Iterative refinement:
  - At each PSO iteration, particle positions are updated.
  - Updated parameters are used to retrain the networks.
  - Retrained models are re-evaluated, and fitness scores are updated accordingly.
3. Stopping criterion: The algorithm stops when a predefined number of PSO iterations is reached or when fitness convergence is observed across the swarm.

This PSO-guided refinement enables coordinated optimization of model parameters across the swarm, while allowing each network to maintain specialization for its designated feature pair through re-training at every iteration. Within this process, the cognitive component encourages each neural network to refine its parameters based on its own historical best configuration, thereby preserving learned patterns specific to its input features. Meanwhile, the social component facilitates knowledge sharing by pulling networks toward the globally best-performing parameter set, promoting convergence toward more effective representations. The inertia weight ω modulates this balance, ensuring a trade-off between the exploration of new parameter spaces and the exploitation of successful configurations. Together, these dynamics allow the swarm to evolve as a collaborative system, where individual networks continuously improve not only through isolated learning but also via collective guidance.

### 2.3 Output integration and final prediction

In the final phase of the PSO-FeatureFusion framework, the outputs from all predictive models are aggregated to produce the final prediction. This step ensures that the model leverages the strengths of multiple feature combinations while mitigating individual model noise or bias (Fig. 1c). Several integration strategies can be employed based on the nature of the prediction task:

1. Regression function: A function (e.g. weighted sum or linear regression) is applied to combine continuous outputs into a single prediction score.
2. Voting mechanism: For classification tasks, a majority vote across models determines the final class label.
3. Averaging: Model outputs are averaged to reduce variance and improve generalization.
4. New model integration: A secondary neural network is trained on the outputs of all base models to learn a higher-level representation and produce the final prediction.

The choice of integration method depends on the problem domain, data characteristics, and prediction objective. By combining multiple model outputs in a principled manner, PSO-FeatureFusion ensures that final predictions are robust, reliable, and reflective of the complex relationships among biological entities. In all experiments reported in this paper, we adopt the New Model Integration strategy. Concretely, once the PSO stage has finished, all base networks (one per feature-pair) are frozen. We then concatenate their outputs and train a lightweight stacking neural network via standard backpropagation to produce the final prediction, and PSO is not applied in this stage.

## 3 Results

This section presents the evaluation of PSO-FeatureFusion on two key bioinformatics tasks: predicting polypharmacy side effects and identifying drug-disease associations. We report the performance of the method on multiple benchmark datasets, assess its generalizability, and compare it against state-of-the-art models using standard evaluation metrics.

### 3.1 Problem description and dataset

To assess the effectiveness of PSO-FeatureFusion, we evaluate it on two distinct bioinformatics problems: predicting polypharmacy side effects and identifying drug-disease associations (DDAs). Each problem is described below, along with the datasets and features used.

Problem 1: Predicting polypharmacy side effects. Unanticipated drug-drug interactions (DDIs) can lead to serious adverse effects, posing risks to patient safety and treatment efficacy. These interactions occur when one drug alters the effect of another during co-administration. Accurate prediction of DDIs is essential for reducing polypharmacy-related risks and optimizing drug combinations. We evaluate this task using three benchmark datasets, DS1, DS2, and DS3, which provide extensive annotations of drug pairs, interaction outcomes, and associated features.

DS1 includes 572 drugs and 37,264 confirmed DDIs, each labeled with one of 65 adverse event types (Deng *et al.* 2020, Al-Rabeah and Lakizadeh 2022). It includes the following drug features:

- Mono Side Effects (9,991 features), from OFFSIDES and SIDER.
- Targets (1,162 features), from DrugBank.
- Enzymes (202 features), from DrugBank.
- Chemical Substructures (881 features), from PubChem.
- Pathways (957 features), from KEGG.

DS2 contains 1258 drugs and 161 770 drug-drug interactions, annotated with 100 adverse event types (Lin *et al.* 2022). Feature sets include:

- Targets (1651 features), from DrugBank.
- Enzymes (316 features), from DrugBank.
- Chemical substructures (2040 features), from PubChem.

DS3, based on the TWOSIDES database (Lin *et al.* 2023, Masumshah and Eslahchi 2023), includes 645 drugs and 63 473 interactions labeled with associated side effects. It provides:

- Mono side effects (10 184 features).
- Targets (8934 features).

Problem 2: Predicting drug-disease associations drug repositioning—identifying new therapeutic uses for existing drugs—can significantly accelerate drug development. Predicting drug-disease associations (DDAs) is a critical step in this process, enabling the discovery of novel therapeutic relationships. We use four datasets for this task: B-Dataset, C-Dataset, F-Dataset, and DDCD.

B-Dataset (Zhang *et al.* 2018) includes 269 drugs, 598 diseases, and 18 416 known DDAs, sourced from the CTD database.

C-Dataset (Luo *et al.* 2016) contains 2532 validated associations among 663 drugs (from DrugBank) and 409 diseases (from OMIM).

F-Dataset (Gottlieb *et al.* 2011) comprises 1933 known DDAs involving 593 drugs and 313 diseases.

DDCD (Hassanali Aragh *et al.* 2024) contains 42 200 drug-disease associations across 1410 drugs and 1573 diseases.

Each dataset is associated with rich feature sets:

- Drug features (common across datasets):
  - Description, targets, pharmacodynamics, SMILES, mechanism, condition, category
- Disease features:
  - B, C, and F datasets: phenotype only.
  - DDCD: description, pathway name, and slim mapping

To facilitate comparison across datasets, we summarize their key characteristics in Table S1, available as supplementary data at *Bioinformatics Advances* online. The table reports the number of drugs, diseases, and interactions/associations in each dataset, along with the feature sets available for drugs and diseases.

### 3.2 Implementation of the PSO-FeatureFusion method

#### 3.2.1 Drug-drug interaction problem

For the DDI task, we applied PSO-FeatureFusion to three datasets. In DS1, each drug was represented by five feature types: targets, enzymes, chemical substructures, pathways, and mono side effects. DS2 included three features per drug, and DS3 included two. To standardize feature dimensions, we computed a Jaccard similarity matrix for each drug feature in every dataset. These matrices captured pairwise similarities between drugs and were used as inputs for combination. The number of resulting feature combinations corresponded to all possible pairwise matches between feature types for a drug pair:

- DS1 →5×5=25 combinations.
- DS2 →3×3=9 combinations.
- DS3 →2×2=4 combinations.

Each combined feature was modeled by a dedicated neural network with identical architecture: three hidden layers (512, 256, 128 neurons) and an output layer sized to match the number of side effect classes in the dataset. All models were trained for 50 epochs. Following training, each neural network was treated as a particle in the PSO algorithm. The goal of PSO was to optimize how the outputs of these models contribute to the final prediction. PSO parameters were:

- Inertia weight ω: 0.7.
- Cognitive and social coefficients $\left(C_{1},C_{2}\right)$: 1.5.
- Number of iterations: 10.

After optimization, outputs from all base models were concatenated and passed to a final neural network with two hidden layers (64 and 32 neurons) and an output layer matching the number of side effect classes. This final model produced the prediction for each drug pair.

#### 3.2.2 DDA problem

For the DDA task, we applied the method to four datasets. In B-dataset, C-dataset, and F-dataset, each drug was described by seven features and each disease by one feature, producing seven combined features per sample. In DDCD, diseases had three features, resulting in 21 combined features per drug-disease pair. As in the DDI task, we computed a similarity matrix for each feature and created combined representations for every drug-disease feature pair. Each was modeled by a neural network with the same architecture: three hidden layers (512, 256, 128 neurons) and a single output neuron with sigmoid activation. All base models were again treated as particles in PSO, using the same parameter settings $(\omega =0.7,C_{1}=C_{2}=1.5,10$ iterations). Finally, outputs from the base models (7 or 21, depending on the dataset) were concatenated and passed to a final neural network with two hidden layers (64 and 32 neurons) and a single sigmoid output, producing the final prediction of a drug-disease association.

### 3.3 Outcome analysis

This section evaluates the performance of PSO-FeatureFusion in comparison to both baseline models and state-of-the-art methods across two bioinformatics problems: drug-drug interaction (DDI) prediction and drug-disease association (DDA) prediction. We use six widely adopted metrics for assessment: accuracy (ACC), area under the precision-recall curve (AUPRC), area under the receiver operating characteristic curve (AUROC), precision, recall, and F-score.

#### 3.3.1 Drug-drug interaction problem

PSO-FeatureFusion is compared with 14 existing methods, including advanced deep learning approaches such as ADEP (adversarial deep ensemble), GGI-DDI (graph-based generative inference), SSF-DDI (sparse subspace features), LSFC (latent space feature combination), DPSP (deep predictive spatial propagation), GNN-DDI (graph neural network), MSTE and MDF-SA-DDI (multisource and deep feature selection), as well as NNPS and DDIMDL (neural network-based frameworks). Traditional machine learning baselines include random forest (RF), K-nearest neighbors (KNN), logistic regression (LR), and decision tree (DT).

DS1 results (Table S2, available as supplementary data at *Bioinformatics Advances* online): PSO-FeatureFusion achieved Precision, Recall, and F-score of 0.9863, and an AUROC of 0.9991, the highest among all models. The method also led in AUPRC (0.9820), outperforming methods like ADEP, SSF-DDI, and DPSP. This demonstrates the method’s superior ability to learn from diverse drug features and accurately model polypharmacy effects.

DS2 results (Table S3, available as supplementary data at *Bioinformatics Advances* online): PSO-FeatureFusion maintained strong performance with Accuracy of 0.9710, AUPRC of 0.9799, and AUROC of 0.9993. Compared to ADEP (AUPRC: 0.9703), it demonstrated a clear performance margin, particularly in precision and recall metrics—both at 0.9709.

DS3 results (Table S4, available as supplementary data at *Bioinformatics Advances* online**)**: The framework again outperformed most baselines, achieving F-score of 0.9212, AUPRC of 0.9680, and AUROC of 0.9899. While ADEP was competitive in accuracy, PSO-FeatureFusion provided more balanced predictions across all evaluation metrics.

**Figure 2.**
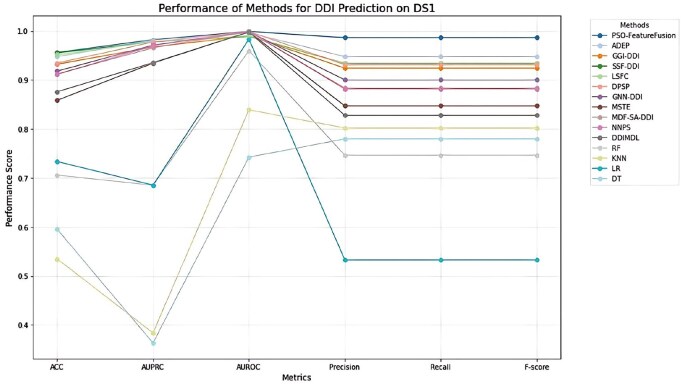
Comparative performance of PSO-FeatureFusion and 14 baseline methods for drug-drug interactions on DS1.

Figure 2, and Figs S1 and S2, available as supplementary data at *Bioinformatics Advances* online, provide visual comparisons of performance across the three DDI datasets, highlighting the consistent strengths of PSO-FeatureFusion across metrics and datasets.

#### 3.3.2 DDA prediction

For DDA prediction, PSO-FeatureFusion is compared with six state-of-the-art methods: MiRAGE, DRHGCN and HINGRL, DRWBNC and DDAGDL, and AMDGT.

Across all datasets (Tables 1 and 2, and Tables S5 and S6, available as supplementary data at *Bioinformatics Advances* online), MiRAGE consistently achieved the highest values in most evaluation metrics, slightly outperforming PSO-FeatureFusion. However, the differences were minimal. For instance, on the B-dataset, MiRAGE obtained an F-score of 0.9917, compared to 0.9747 for PSO-FeatureFusion. On the C-dataset, the F-scores were 0.9214 for MiRAGE and 0.9205 for PSO-FeatureFusion. Similarly, on the F-dataset, MiRAGE achieved 0.9907 versus 0.9431, and on the DDCD dataset, 0.9920 versus 0.9917. These results indicate that while MiRAGE had a slight edge in predictive performance, PSO-FeatureFusion remained highly competitive, with consistently strong results across all datasets.

**Table 1.** Comparison of PSO-FeatureFusion with other methods for DDA prediction on B-dataset.

Method	ACC	AUPRC	AUROC	Precision	Recall	F-score
PSO-FeatureFusion	0.9751	0.9750	0.9751	0.9888	0.9611	0.9747
MiRAGE	**0.9918**	**0.9996**	**0.9996**	**0.9917**	**0.9918**	**0.9917**
DRHGCN	0.8268	0.9106	0.9092	0.8678	0.7711	0.8166
HINGRL	0.8035	0.8774	0.8845	0.8006	0.8084	0.8045
DRWBNC	0.5991	0.9018	0.9004	0.9810	0.2021	0.3352
DDAGDL	0.7646	0.8315	0.8421	0.7616	0.7703	0.7659
AMDGT	0.8629	0.9309	0.9337	0.8614	0.8650	0.8632

Bold values indicate the highest performance for each metric, while underlined values indicate the second highest performance.

**Table 2.** Comparison of PSO-FeatureFusion with other methods for DDA prediction on DDCD dataset.

Method	ACC	AUPRC	AUROC	Precision	Recall	F-score
PSO-FeatureFusion	0.9974	0.9980	0.9899	0.9992	0.9884	0.9917
RF	**0.9997**	**0.9994**	**0.9998**	**0.9998**	**0.9921**	**0.9920**
LR	0.9947	0.8877	0.9749	0.9952	0.8913	0.8566
KNN	0.9960	0.8561	0.9343	0.9944	0.9039	0.8908
DT	0.9961	0.8350	0.8840	0.9991	0.9884	0.9113
MLP-1	0.9969	0.9229	0.9867	0.9883	0.9318	0.9175
MLP-2	0.9969	0.9265	0.9888	0.9903	0.9304	0.9187

Bold values indicate the highest performance for each metric, while underlined values indicate the second highest performance.

Moreover, PSO-FeatureFusion clearly outperformed the other five baseline methods—DRHGCN, HINGRL, DRWBNC, DDAGDL, and AMDGT—in terms of overall balance, recall, and precision. This superiority was especially evident in datasets with complex graph structures, where PSO-FeatureFusion achieved more stable and reliable predictions.

An important distinction lies in computational efficiency. While MiRAGE achieves slightly higher predictive performance, it relies on a more complex and computationally intensive architecture, resulting in longer training and inference times. In contrast, PSO-FeatureFusion significantly reduces computational cost due to its streamlined feature integration strategy. Experimental benchmarks showed that PSO-FeatureFusion required substantially less time to train and evaluate across all datasets. This makes it a more practical and scalable choice for large-scale or time-sensitive biomedical applications, where efficiency is a critical factor.

We evaluated the computational efficiency of PSO-FeatureFusion in comparison with the baseline methods. The comparison was conducted using all datasets under identical hardware and software conditions (NVIDIA RTX 3080 GPU, batch size = 128, 50 epochs). For the DDI task, PSO-FeatureFusion consistently demonstrated both higher predictive performance and shorter training and evaluation times compared to the baseline methods. This advantage stems from its modular architecture, which enables parallel training of lightweight neural networks and efficient inference. For the DDA task, a detailed runtime comparison with MiRAGE is provided in Table 3, and Tables S7 and S8, available as supplementary data at *Bioinformatics Advances* online. While MiRAGE achieves slightly higher predictive accuracy, PSO-FeatureFusion requires substantially less computational time, highlighting a favorable balance between efficiency and accuracy. Overall, these results indicate that PSO-FeatureFusion provides a strong trade-off between computational cost and predictive accuracy across both tasks, making it a practical choice for large-scale bioinformatics applications.

**Table 3.** Runtime comparison of PSO-FeatureFusion and MiRAGE on the B-Dataset for the DDA task.

Method	Training time (min)	Evaluation time (sec/sample)
PSO-FeatureFusion	**50**	**0.14**
MiRAGE	113	0.40

Bold values indicate the best (lowest) runtime for each method.

Figures S3–S6, available as supplementary data at *Bioinformatics Advances* online, visualize the comparative performance across datasets, further illustrating PSO-FeatureFusion’s strengths in recall, precision, and F-score—critical metrics for minimizing false negatives in drug discovery workflows.

Overall, the results demonstrate that PSO-FeatureFusion consistently delivers competitive or superior performance across both DDI and DDA prediction tasks. In DDI prediction, it achieved top scores in F-score, AUROC, and AUPRC across all three datasets, surpassing or matching advanced graph-based and ensemble learning models. In the DDA task, PSO-FeatureFusion remained highly competitive with state-of-the-art methods like MiRAGE, achieving strong precision-recall balance and outperforming many baselines in F-score and robustness. These results confirm the framework’s ability to integrate heterogeneous features, model complex relationships, and generalize effectively across multiple bioinformatics problems. Across all datasets, PSO-FeatureFusion consistently achieved superior performance compared to baseline methods. For instance, on DS1 our framework achieved an AUROC of 0.9991, which is higher than the second-best method DPSP (0.9990). On DS2, PSO-FeatureFusion obtained an AUPRC of 0.9799, outperforming ADEP (0.9703) by 0.96%. On the DDCD dataset, PSO-FeatureFusion reached an F-score of 0.9917, close to that of the competitor RF while offering a more balanced trade-off between precision and recall. These differences, though sometimes small in absolute value, are consistent across all datasets and metrics, demonstrating the robustness of the proposed method.

## 4 Discussion

The proposed PSO-FeatureFusion framework offers a generalizable and effective strategy for modeling complex biological relationships by integrating heterogeneous features through particle swarm optimization. Unlike conventional methods that often use fixed or independent fusion mechanisms, PSO-FeatureFusion dynamically learns how to combine pairwise feature interactions, enabling the creation of more expressive and task-specific representations. The superiority of PSO-FeatureFusion over existing approaches can be attributed to its dynamic and adaptive feature integration mechanism. Unlike similarity-based or matrix factorization methods, which rely on static feature representations, our approach uses PSO to optimize the relative contributions of feature pairs, thereby capturing complex interdependencies between heterogeneous biological features. Compared to deep learning-based fusion strategies, PSO-FeatureFusion avoids heavy end-to-end training and provides a more efficient search in the integration space, which reduces computational cost while maintaining high predictive performance. This combination of adaptability, efficiency, and interpretability emphasizes the novelty of our framework relative to prior approaches. To evaluate the framework, we applied it to two important bioinformatics tasks: drug-drug interaction (DDI) prediction and drug-disease association (DDA) prediction, using multiple datasets with diverse structures and feature compositions. In the DDI task, datasets included three to five drug features, resulting in 9 to 25 pairwise feature combinations, each modeled by a dedicated neural network. PSO was then applied to optimize the contributions of these networks in the final prediction. Although the individual feature pairs differ in biological interpretation, all input vectors are dimensionally standardized, and all models share a common structure. The PSO process enhances ensemble performance by optimizing feature-pair contributions rather than enforcing global parameter alignment. The convergence of PSO has been extensively studied, and it is well established that with appropriate parameter settings (e.g. inertia weight ω and acceleration coefficients *c*1 and *c*2), the algorithm achieves stable convergence in high-dimensional optimization problems. As a stochastic optimizer, PSO iteratively updates particle positions and velocities, gradually guiding the swarm toward the global optimum. In our experiments, the optimization process consistently stabilized after a limited number of iterations (typically fewer than 50), demonstrating reliable convergence without oscillations or divergence. Furthermore, in our framework, PSO is employed to optimize the integration weights of feature-pair models rather than to train the neural networks directly. This design keeps the optimization space relatively constrained, which further facilitates convergence and ensures computational efficiency. The results demonstrated strong and consistent performance across classification metrics, particularly Precision, Recall, and F-score. Although PSO-FeatureFusion achieved high performance metrics, these results stem from its design and optimization strategy. Other strong methods also achieved high accuracy, and in some datasets (e.g. DS3, C-Dataset), the task remained challenging. Thus, the reported results reflect the robustness of our framework, not the simplicity of the underlying problems. The fact that PSO-FeatureFusion does not always achieve the single best score in every dataset is expected, as different models may exploit particular feature structures more effectively in specific settings. However, the main advantage of our framework lies in its ability to achieve consistently high and balanced performance across all metrics and datasets. In particular, PSO-FeatureFusion provides a favorable trade-off between precision and recall, which is especially critical in bioinformatics applications where false negatives must be minimized. Furthermore, unlike many deep learning-based approaches, our method achieves these results with greater computational efficiency and scalability, reinforcing its practical utility and generalizability. For DDA prediction, PSO-FeatureFusion was evaluated on four datasets with varying levels of complexity. In three datasets, each drug had seven features while each disease had one, resulting in seven combinations; the fourth dataset included three disease features, increasing the total to 21 combinations. The framework maintained its effectiveness in this setting, achieving high recall and F-score values—crucial in drug discovery scenarios where reducing false negatives is especially important. Although PSO-FeatureFusion is currently applied to two entity groups, the architecture is naturally extensible to scenarios involving three or more biological entity types. In such cases, feature triplets (or higher-order combinations) can be modeled individually and integrated through a generalized PSO fusion mechanism, enabling flexible and scalable multientity prediction. One of the key strengths of PSO-FeatureFusion is its efficiency in constrained optimization settings. Despite using a limited number of PSO iterations, the method consistently delivered high performance, demonstrating robustness even without extensive hyperparameter tuning. This makes the framework practical for researchers with limited computational resources or those working with large-scale biological data. Additionally, the modular structure of the framework—assigning a distinct model to each feature pair—makes it highly parallelizable and scalable. With appropriate hardware, the model training phase can be distributed across computing nodes, allowing PSO-FeatureFusion to be deployed on larger datasets or integrated into high-throughput pipelines. The framework is also designed for practical usability. It avoids reliance on handcrafted similarity metrics, does not require task-specific architecture changes, and can be directly applied to a wide range of biological problems. This plug-and-play design lowers the barrier to entry for researchers who may lack deep expertise in machine learning, while still enabling strong predictive performance. In addition, the pairwise modeling approach supports a degree of interpretability by isolating the predictive contribution of specific feature combinations. This can help researchers identify which biological features—and which interactions between them—are most informative for a given task, offering both predictive power and biological insight. Nonetheless, certain limitations remain. The current framework relies on precomputed similarity matrices, which may not capture nonlinear or semantic relationships between features. Moreover, only pairwise interactions are modeled—higher-order combinations and temporal relationships are not yet explored. Future extensions may incorporate graph neural networks, attention mechanisms, or differentiable fusion layers to address these limitations and enhance both expressiveness and flexibility. In summary, PSO-FeatureFusion provides a flexible, high-performing, and interpretable modeling framework for bioinformatics. Its ability to generalize across tasks and datasets, while preserving feature individuality and optimizing interaction modeling, makes it a strong foundation for future research in areas such as drug repositioning, adverse effect prediction, and precision medicine.

## 5 Conclusion

This study presents PSO-FeatureFusion, a novel framework for integrating heterogeneous biological features using particle swarm optimization. By jointly modeling pairwise feature interactions and optimizing their contributions, the method addresses key challenges in bioinformatics, including feature heterogeneity, dimensional mismatch, and data sparsity. Evaluated on two representative tasks, PSO-FeatureFusion demonstrated strong predictive performance and adaptability across diverse datasets. Its task-agnostic design and modular structure enable broad applicability to a wide range of biological problems, making it a practical and scalable solution for real-world bioinformatics applications. Looking ahead, PSO-FeatureFusion offers a flexible foundation for further research. Future extensions may incorporate more expressive fusion strategies, temporal dynamics, or graph-based architectures to further enhance performance and interpretability in complex biological systems. In addition, incorporating more advanced machine learning techniques could further enhance the predictive power of bioinformatics models. To highlight this potential, we have cited the recommended references (Yang *et al.* 2025a) and (Yang *et al.* 2025b) as promising directions for future research.

## Supplementary Material

vbaf263_Supplementary_Data
