# Supplementary material for: PSO-FeatureFusion: a general framework for fusing heterogeneous features via particle swarm optimization
Source: Bioinform Adv. 2025 Oct 29;5(1):vbaf263. doi: 10.1093/bioadv/vbaf263 (PMC12596698; doi:10.1093/bioadv/vbaf263)
Supplement: vbaf263_Supplementary_Data [file vbaf263_supplementary_data.pdf]

Table S1. Overview of Datasets

Dataset	Task	No. Drugs	No. Diseases	No. Interactions / Associations	No. Features per Drug	No. Features per Disease	Feature Types
DS1	DDI	572	–	37,264 interactions / 65 side effect types	5 (Mono side effects, Targets, Enzymes, Chemical substructures, Pathways)	–	Drug features
DS2	DDI	1,258	–	161,770 interactions / 100 side effect types	3 (Targets, Enzymes, Chemical substructures)	–	Drug features
DS3	DDI	645	–	63,473 interactions	2 (Mono side effects, Targets)	–	Drug features
B-Dataset	DDA	269	598	18,416 associations	7 (Description, Targets, Pharmacodynamics, SMILES, Mechanism, Condition, Category)	1 (Phenotype)	Drug & disease features
C-Dataset	DDA	663	409	2,532 associations	7 (as above)	1 (Phenotype)	Drug & disease features
F-Dataset	DDA	593	313	1,933 associations	7 (as above)	1 (Phenotype)	Drug & disease features
DDCD	DDA	1,410	1,573	42,200 associations	7 (as above)	3 (Description, Pathway name, Slim mapping)	Drug & disease features

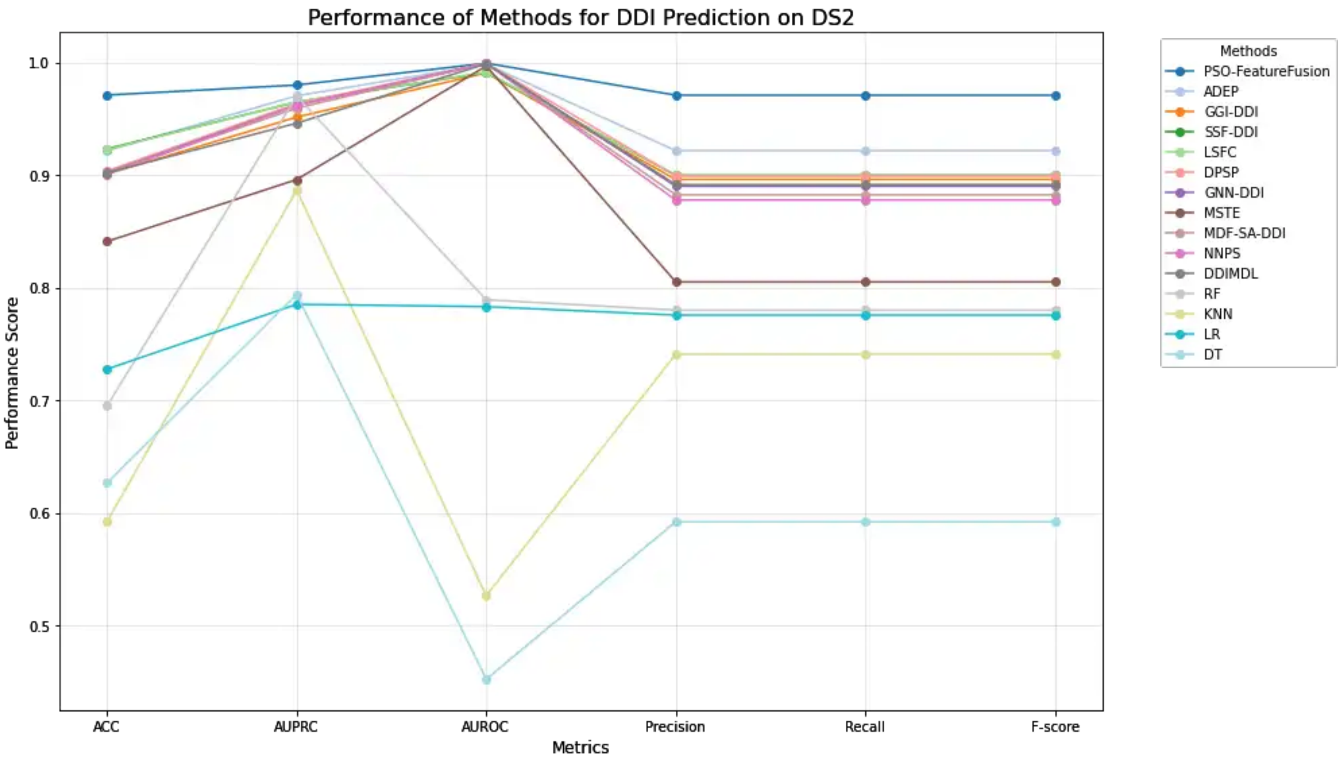

Fig. S1. Comparative performance of PSO-FeatureFusion and 14 baseline methods for drug-drug interactions on DS2.

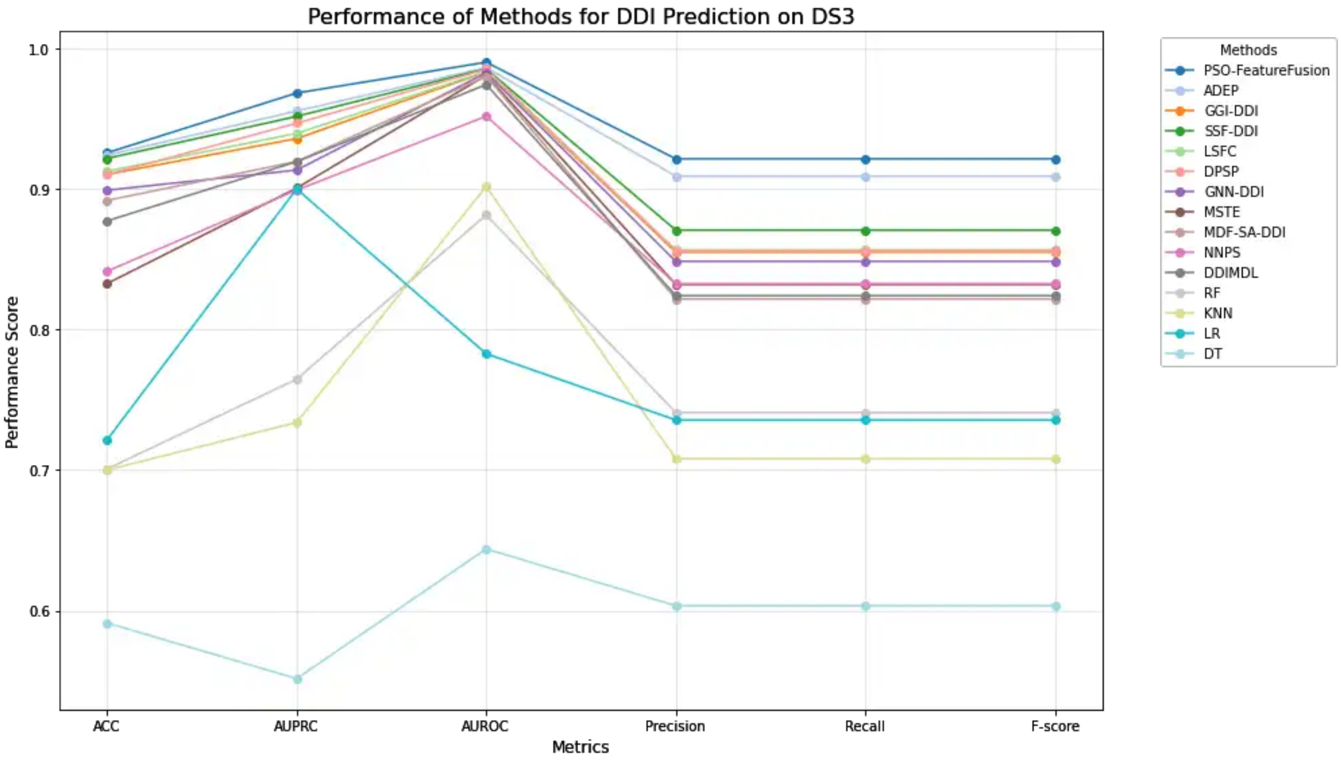

Fig. S2. Comparative performance of PSO-FeatureFusion and 14 baseline methods for drug-drug interactions on DS3.

Table S2. Comparison of PSO-FeatureFusion with other methods for DDI prediction on DS1

Method	ACC	AUPRC	AUROC	Precision	Recall	F-score
PSO-FeatureFusion	**0.9560**	**0.9820**	**0.9991**	**0.9863**	**0.9863**	**0.9863**
ADEP	0.9476	0.9811	0.9963	0.9476	0.9476	0.9476
GGI-DDI	0.9320	0.9672	0.9900	0.9242	0.9242	0.9242
SSF-DDI	0.9552	0.9777	0.9890	0.9337	0.9337	0.9337
LSFC	0.9514	0.9780	0.9901	0.9339	0.9339	0.9339
DPSP	0.9344	0.9773	0.9990	0.9309	0.9309	0.9309
GNN-DDI	0.9180	0.9709	0.9985	0.8999	0.8999	0.8999
MSTE	0.8584	0.9343	0.9981	0.8470	0.8470	0.8470
MDF-SA-DDI	0.9121	0.9657	0.9989	0.8832	0.8832	0.8832
NNPS	0.9113	0.9699	0.9989	0.8815	0.8815	0.8815
DDIMDL	0.8757	0.9353	0.9977	0.8277	0.8277	0.8277
RF	0.7058	0.6850	0.9596	0.7463	0.7463	0.7463
KNN	0.5350	0.3836	0.8390	0.8019	0.8019	0.8019
LR	0.7336	0.6853	0.9831	0.5327	0.5327	0.5327
DT	0.5953	0.3631	0.7423	0.7796	0.7796	0.7796

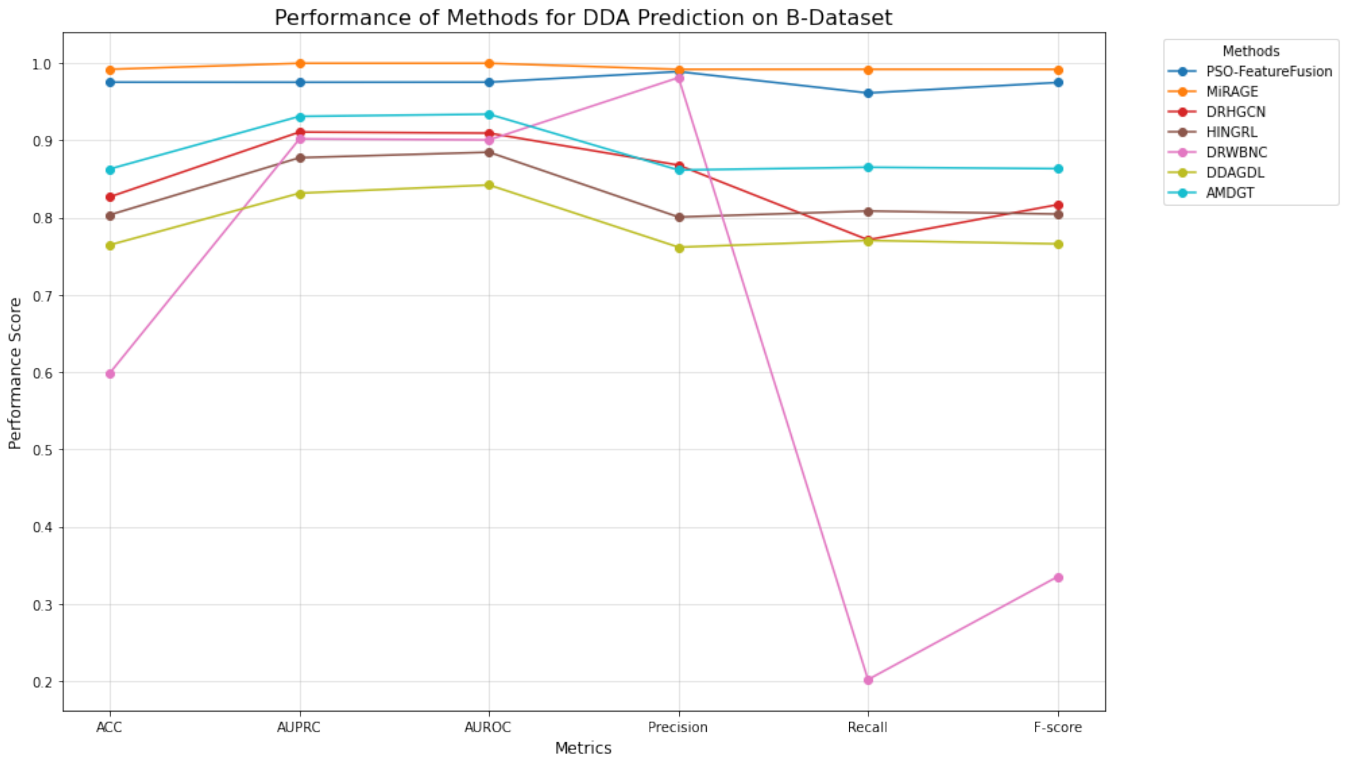

Fig. S3. Performance comparison of PSO-FeatureFusion and six benchmark models for drug-disease associations on B-Dataset.

Table S3. Comparison of PSO-FeatureFusion with other methods for DDI prediction on DS2

Method	ACC	AUPRC	AUROC	Precision	Recall	F-score
PSO-FeatureFusion	**0.9710**	**0.9799**	**0.9993**	**0.9709**	**0.9709**	**0.9709**
ADEP	0.9216	0.9703	0.9983	0.9216	0.9216	0.9216
GGI-DDI	0.9009	0.9514	0.9906	0.8960	0.8960	0.8960
SSF-DDI	0.9232	0.9648	0.9910	0.8997	0.8997	0.8997
LSFC	0.9226	0.9650	0.9913	0.8998	0.8998	0.8998
DPSP	0.9036	0.9633	0.9993	0.8990	0.8990	0.8990
GNN-DDI	0.9020	0.9619	0.9991	0.8900	0.8900	0.8900
MSTE	0.8409	0.8959	0.9968	0.8050	0.8050	0.8050
MDF-SA-DDI	0.9018	0.9593	0.9991	0.8824	0.8824	0.8824
NNPS	0.9006	0.9616	0.9990	0.8777	0.8777	0.8777
DDIMDL	0.9019	0.9460	0.9987	0.8915	0.8915	0.8915
RF	0.6948	0.9700	0.7891	0.7799	0.7799	0.7799
KNN	0.5923	0.8866	0.5262	0.7408	0.7408	0.7408
LR	0.7275	0.7851	0.7830	0.7755	0.7755	0.7755
DT	0.6265	0.7939	0.4521	0.5920	0.5920	0.5920

Table S4. Comparison of PSO-FeatureFusion with other methods for DDI prediction on DS3

Method	ACC	AUPRC	AUROC	Precision	Recall	F-score
PSO-FeatureFusion	**0.9255**	**0.9680**	**0.9899**	**0.9212**	**0.9212**	**0.9212**
ADEP	0.9238	0.9552	0.9861	0.9089	0.9089	0.9089
GGI-DDI	0.9101	0.9356	0.9830	0.8549	0.8549	0.8549
SSF-DDI	0.9215	0.9514	0.9852	0.8705	0.8705	0.8705
LSFC	0.9124	0.9393	0.9833	0.8564	0.8564	0.8564
DPSP	0.9100	0.9465	0.9849	0.8558	0.8558	0.8558
GNN-DDI	0.8989	0.9132	0.9824	0.8483	0.8483	0.8483
MSTE	0.8325	0.9004	0.9806	0.8319	0.8319	0.8319
MDF-SA-DDI	0.8916	0.9193	0.9799	0.8215	0.8215	0.8215
NNPS	0.8413	0.8990	0.9515	0.8324	0.8324	0.8324
DDIMDL	0.8771	0.9192	0.9741	0.8239	0.8239	0.8239
RF	0.7007	0.7645	0.8814	0.7408	0.7408	0.7408
KNN	0.6999	0.7338	0.9015	0.7079	0.7079	0.7079
LR	0.7214	0.8998	0.7826	0.7355	0.7355	0.7355
DT	0.5912	0.5516	0.6438	0.6034	0.6034	0.6034

Table S5. Comparison of PSO-FeatureFusion with other methods for DDA prediction on C-Dataset

**Method**	**ACC**	**AUPRC**	**AUROC**	**Precision**	**Recall**	**F-score**
PSO-FeatureFusion	0.9193	0.9300	0.9290	0.9209	0.9202	0.9205
MiRAGE	**0.9209**	**0.9672**	**0.9733**	**0.9210**	**0.9209**	**0.9214**
DRHGCN	0.8652	0.9427	0.9324	0.9192	0.8008	0.8559
HINGRL	0.8698	0.9457	0.9372	0.8851	0.8500	0.8672
DRWBNC	0.8663	0.9419	0.9234	0.8984	0.8370	0.8612
DDAGDL	0.8168	0.8935	0.8693	0.7874	0.7721	0.7797
AMDGT	0.9062	0.9698	0.9681	0.8903	0.9265	0.9081

Table S6. Comparison of PSO-FeatureFusion with other methods for DDA prediction on F-Dataset

**Method**	**ACC**	**AUPRC**	**AUROC**	**Precision**	**Recall**	**F-score**
PSO-FeatureFusion	0.9454	0.9736	0.9445	0.9593	0.9275	0.9431
MiRAGE	**0.9920**	**0.9985**	**0.9985**	**0.9930**	**0.9907**	**0.9907**
DRHGCN	0.8583	0.9375	0.9207	0.9309	0.7739	0.8452
HINGRL	0.8645	0.9449	0.9366	0.8832	0.8402	0.8612
DRWBNC	0.8296	0.9200	0.8958	0.8752	0.8237	0.8341
DDAGDL	0.8513	0.9235	0.9239	0.8475	0.8567	0.8521
AMDGT	0.8905	0.9617	0.9598	0.8741	0.9128	0.8929

Table S7. Runtime comparison of PSO-FeatureFusion and MiRAGE on the C-Dataset for the DDA task

**Method**	**Training Time (min)**	**Evaluation Time (sec/sample)**
PSO-FeatureFusion	**52**	**0.15**
MiRAGE	110	0.40

Table S8. Runtime comparison of PSO-FeatureFusion and MiRAGE on the F-Dataset for the DDA task

**Method**	**Training Time (min)**	**Evaluation Time (sec/sample)**
PSO-FeatureFusion	**54**	**0.16**
MiRAGE	109	0.40

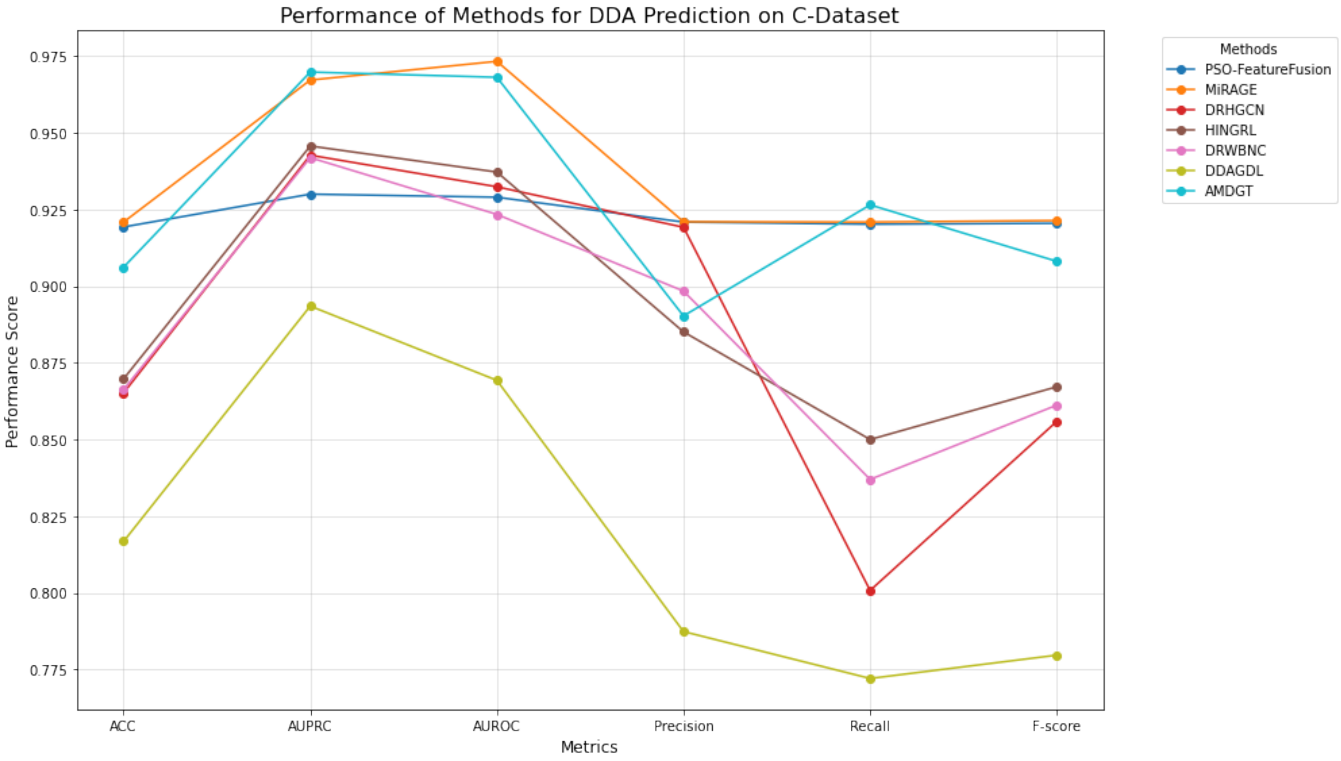

Fig. S4. Performance comparison of PSO-FeatureFusion and six benchmark models for drug-disease associations on C-Dataset.

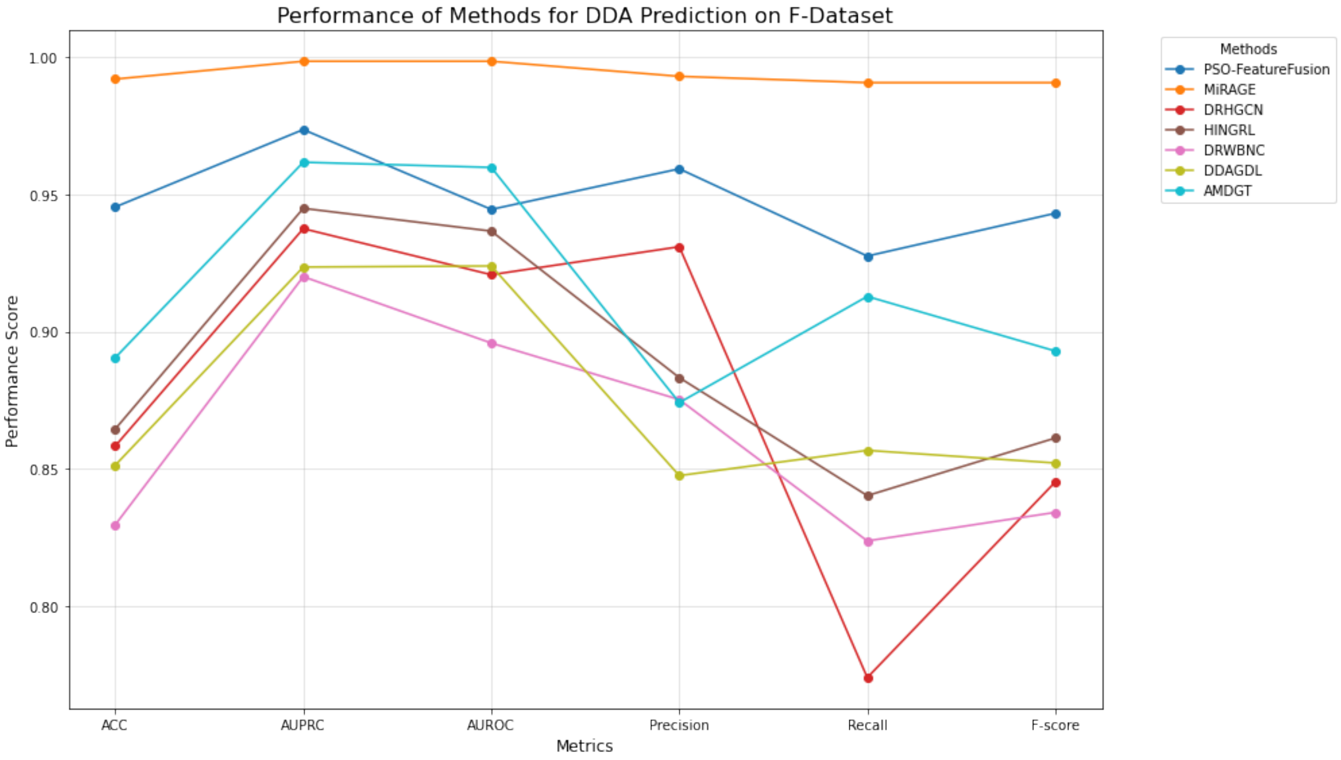

Fig. S5. Performance comparison of PSO-FeatureFusion and six benchmark models for drug-disease associations on F-Dataset.

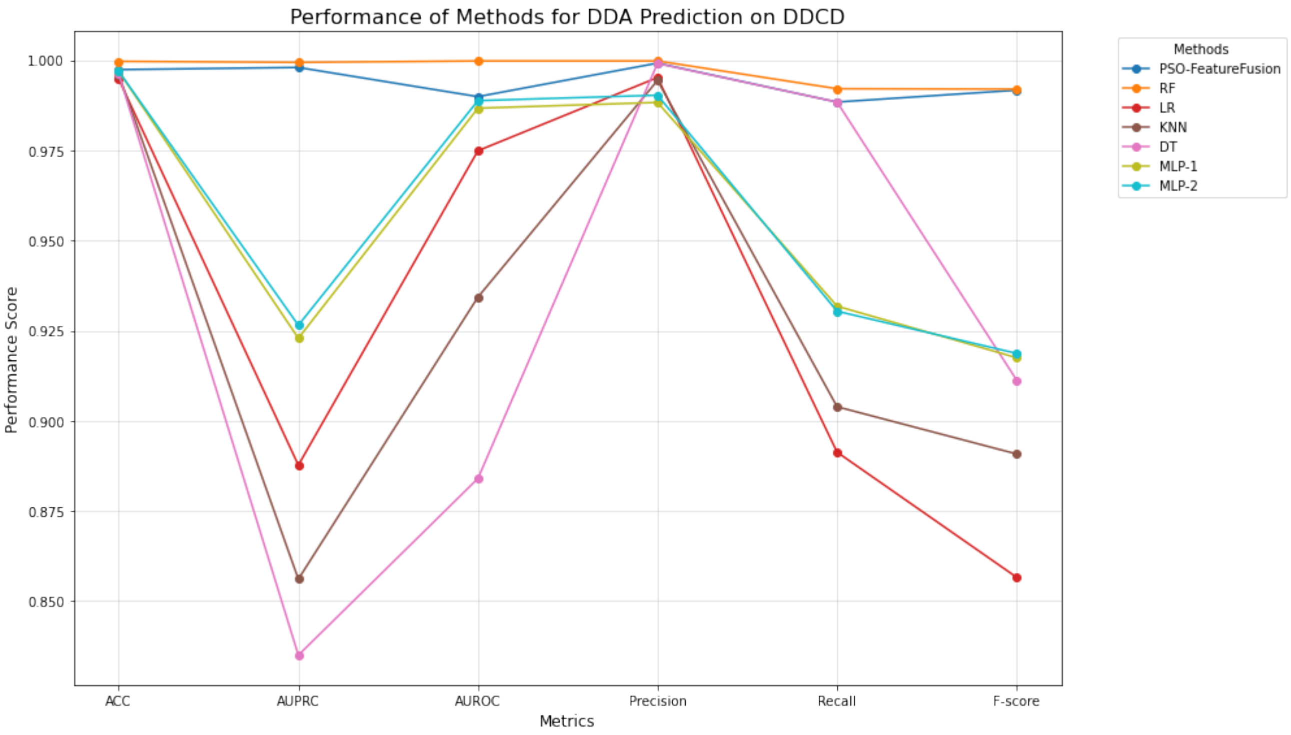

Fig. S6. Performance comparison of PSO-FeatureFusion and six benchmark models for drug-disease associations on DDCD.
